# ATPase inhibitory factor 1 is a potential prognostic marker for the migration and invasion of glioma

**DOI:** 10.3892/ol.2015.3548

**Published:** 2015-07-30

**Authors:** JIANHENG WU, QIAO SHAN, PEIDONG LI, YUEHUI WU, JINGWEI XIE, XINJUN WANG

**Affiliations:** Department of Neurosurgery, The Fifth Affiliated Hospital of Zhengzhou University, Zhengzhou, Henan 450052, P.R. China

**Keywords:** adenosine triphosphatase inhibitory factor 1, glioma, prognosis, migration, invasion

## Abstract

Adenosine triphosphatase inhibitory factor 1 (IF1) has previously been considered to be a driving oncogene in human cancers. Several studies have revealed that IF1 overexpression is present in a variety of tumor types and promotes tumor growth and metastasis. The present study aimed to investigate the clinical significance of IF1 in glioma and the role of IF1 in cell migration and invasion. The mRNA and protein expression of IF1 in glioma tissues was found to be significantly increased compared with the expression in normal brain tissues (P<0.05). The presence of IF1 expression was significantly associated with an advanced clinical stage in glioma (P<0.05). Furthermore, the presence of IF1 expression was found to be associated with a reduced overall survival rate of glioma patients (P<0.05). Multivariate Cox regression analysis indicated that IF1 was an independent factor for predicting the overall survival rate of patients with glioma (P<0.05). IF1 knockdown also significantly reduced the number of migratory and invasive U251 and U87 cells (P<0.05). In addition, IF1 knockdown inhibited the expression of nuclear factor-κB (NF-κB) and Snai1, and led to increased E-cadherin expression and reduced vimentin expression. In conclusion, the presence of IF1 expression is associated with poor clinicopathological features in glioma. IF1 expression is an independent prognostic marker for predicting the overall survival rate of patients with glioma. Mechanistically, IF1 may promote glioma cell migration and invasion through the NF-κB/Snai1 axis.

## Introduction

Malignant glioma is the most common malignant tumor that occurs in the brain (1). The World Health Organization (WHO) classification system is used for tumor grading, and the prognosis and management of the disease are indicated by the tumor classification (2). Tumor resection followed by radiotherapy and temozolomide treatment is the current standard therapy for glioma. However, the majority of patients with glioma exhibit tumor progression within two years of diagnosis (3). Therefore, it is important to develop a potent prognostic marker and therapeutic target for human glioma.

Downregulation of oxidative phosphorylation in combination with the activation of aerobic glycolysis is a hallmark for numerous types of human cancer (4–6). H^+^-adenosine triphosphate (ATP) synthase acts as a critical marker for energy metabolism and cell fate. ATPase inhibitory factor 1 (IF1) is a heat-stable protein in mammals that is mainly located within the mitochondrial matrix (7). IF1 has been considered to be an inhibitor for the activity of the mitochondrial H^+^-ATP synthase (8). Elevated IF1 expression is observed in a number of human cancers, including colon (9), lung (10), breast (10) and ovarian cancers (10) and hepatocellular carcinoma (HCC) (11). A previous study have reported that reciprocal activation between IF1 and nuclear factor (NF)-κB promoted HCC angiogenesis and metastasis (11). In colon cancer cells, IF1 promoted aerobic glycolysis and reactive oxygen species-mediated signaling pathway to enhance cell proliferation and cell survival (9). Furthermore, IF1 has been considered to be an independent prognostic marker for human cancer (11). However, the clinical significance of IF1 and its role in glioma metastasis have been insufficiently investigated.

In the present study, IF1 expression was detected in human glioma tissues using immunohistochemical staining and reverse transcription-quantitative polymerase chain reaction (RT-qPCR). The association between IF1 expression and the clinicopathological features of glioma were systemically analyzed. Furthermore, the role of IF1 in the migration and invasion of glioma cells was investigated to confirm the effect of IF1 on the initiation and development of human glioma.

## Materials and methods

### 

#### Clinical samples

A total of 86 paraffin-embedded glioma and 20 normal brain (NB) tissue samples were obtained from the Fifth Affiliated Hospital of Zhengzhou University (Zhengzhou, Henan, China) between January 2008 and December 2010. All samples were used subsequent to obtaining informed consent from patients. The demographical features and clinicopathological data of the patients are reported in Table I. The diagnosis of all specimens was pathologically confirmed and the tissues were classified according to the WHO criteria. The Zhengzhou University Ethics Committee approved all protocols, which were in accordance with the Declaration of Helsinki (12).

#### Immunohistochemical staining

Immunohistochemistry with streptavidin peroxidase conjugate was performed using formalin-fixed paraffin-embedded sections. The sections underwent dewaxing, rehydration, antigen retrieval, endogenous peroxidase activity blocking and goat serum blocking, and the sections were then incubated with the mouse anti-human IF1 monoclonal antibody (clone no., 5E2D7; catalog no., ab110277; Abcam, Cambridge, MA, USA) at 4°C overnight. SP-conjugated secondary antibody and 3,3′-diaminobenzidine were used for the staining of sections. According to the percentage of positive tumor cells, IF1 expression was classified as absent (<10%) or present (≥10%) (13).

#### RT-qPCR

The IF1 primers used were as follows: Forward, 5′-GGGCCTTCGGAAAGAGAG-3′ and reverse, 5′-TTCAAA GCTGCCAGTTGTTC-3′. PCR amplification was performed to quantify the expression of IF1 and GAPDH mRNA using a SYBR Premix Ex Taq ii (Perfect Real Time) kit (Takara Bio, Otsu, Shiga, Japan), as previously described (14).

#### Cell lines and transfection

The human glioma U251 and U87 cell lines (Chinese Academy of Sciences, Shanghai, China) were cultured in complete Dulbecco's modified Eagle medium (DMEM; Gibco Life Technologies, Grand Island, NY, USA) containing 10% fetal bovine serum (FBS; Gibco Life Technologies), 100 U/ml penicillin and 100 µg/ml streptomycin (Sigma-Aldrich, St. Louis, MO, USA) at 37°C in an incubator with a humidified atmosphere containing 5% CO_2_.

Short hairpin (sh)RNA, consisting of IF1 shRNA and non-targeting (NT) shRNA, was purchased from GeneCopoeia, Inc. (Rockville, MD, USA). The cells were transfected with the aforementioned vectors using Lipofectamine 2000 (Invitrogen, Carlsbad, CA, USA), according to the manufacturer's instructions.

#### Western blot analysis

Rabbit anti-human NF-κB p50 polyclonal antibodies (1:1,000 dilution; catalog no., 06-886; Millipore, Billerica, MA, USA), mouse anti-human Snai1 monoclonal antibodies (1:1,000 dilution; catalog no., ab117866; Abcam), rabbit anti-human vimentin polyclonal antibodies (1:1,000 dilution; catalog no., 3932; Cell Signaling Technology, Danvers, MA, USA), rabbit anti-human E-cadherin monoclonal antibodies (1:1,000 dilution; catalog no., 3195; Cell Signaling), mouse anti-human IF1 monoclonal antibodies (1:1,000 dilution; catalog no., ab110277; Abcam) and mouse anti-human anti-GAPDH monoclonal antibodies (1:5,000 dilution; catalog no., G8140-01V; US Biological, Salem, MA, USA) were used for the immunoblotting assay. Horseradish peroxidase-conjugated sheep anti-mouse secondary antibody (Bio-Rad Laboratories, Hercules, CA, USA) was used at a 1:1,000-1:5,000 dilution and detected by a western blotting Luminol reagent (catalog no., sc-2048; Santa Cruz Biotechnology, Inc., Dallas, TX, USA) (14).

#### Transwell cell migration and invasion assays

Transwell cell migration assays were performed on 12 well plates with 8-µm BioCoat control inserts (Becton-Dickinson, Franklin Lakes, NJ, USA). In total, 1–2×10^5^ U251 or U87 cells transfected with IF1 or NT shRNA were suspended in 500 µl serum-free DMEM and then seeded in the upper well of a Transwell chamber. DMEM (750 µl) supplemented with 10% FBS was added to the lower well. Subsequent to completion, the membranes were removed, the cells on the side facing the upper well were wiped with a cotton swab, and the adherent cells on the undersurface of the insert were stained using crystal violet. At least six representative images of each well were captured and the number of cells were counted using ImageJ software. The BioCoat Matrigel invasion chamber (Becton Dickinson Labware) was used for Transwell cell invasion assays and the following protocols were the same as the Transwell cell migration assays. The experiments were performed in triplicate (15).

#### Statistical analysis

The results were expressed as the mean ± standard error of the mean. The data was analyzed using the SPSS statistical package for Windows version 13 (SPSS, Chicago, IL, USA) or GraphPad Prism 5 software (GraphPad Software, Inc., San Diego, CA, USA). Pearson's χ^2^ test, Kaplan-Meier analysis, log-rank test, multivariate Cox regression analysis or a two-tailed Student's *t*-test, when appropriate. P<0.05 was considered to indicate a statistically significant difference.

## Results

### 

#### Expression of IF1 in glioma and NB tissues

**Figure 1.**
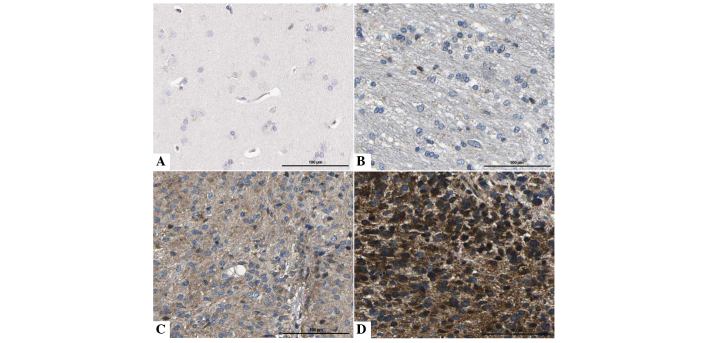
Immunohistochemical staining of IF1 in glioma and NB tissues. IF1 was localized within the cytoplasm. (A) NB tissues without IF1 expression. (B) Low, (C) medium and (D) high expression of IF1 in the tumor cells of glioma tissue compared with (A). Scale bar, 100 µm. NB, normal brain; IF1, adenosine triphosphatase inhibitory factor 1.

To determine the expression of IF1 in glioma specimens, the levels of IF1 expression were detected in 86 glioma and 20 NB tissue specimens using immunohistochemical staining. IF1 expression was considered as either absent or present. The current data indicated that IF1 expression was present in 68.6% (59/86) of glioma tissues, while only 20.0% (4/20) of NB tissues exhibited a signal for IF1 expression (P<0.05; Fig. 1). Furthermore, qPCR was performed to determine the levels of IF1 mRNA in glioma tissues (n=20) and NB tissues (n=20). Quantitative analysis indicated that the level of IF1 mRNA in glioma tissues was significantly increased compared with the level in NB tissues (P<0.05).

#### Clinical significance of IF1 in glioma

**Figure 2.**
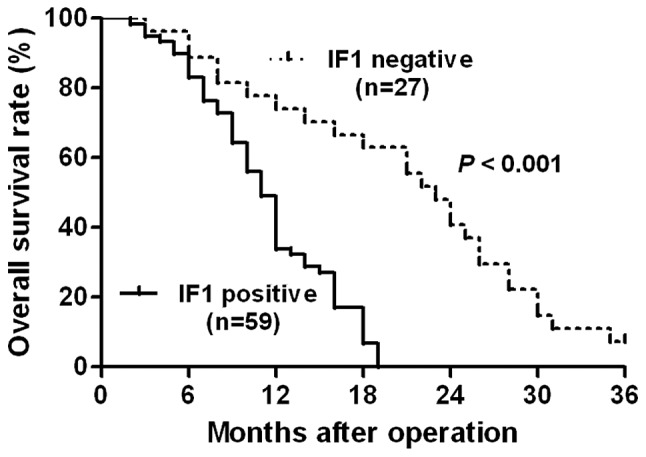
Kaplan-Meier survival analysis of the overall survival rate of 86 glioma patients, according to IF1 protein expression. The expression of IF1 conferred an unfavorable survival rate for glioma patients. IF1, adenosine triphosphatase inhibitory factor 1.

To investigate the clinical significance of IF1 in glioma, the association between IF1 expression and clinicopathological parameters in glioma was investigated. As reported in Table I, the clinical association analysis performed using Pearson's χ^2^ test indicated that the presence of IF1 expression was evidently associated with an advanced clinical stage (P=0.003). Furthermore, Kaplan-Meier analysis indicated that tumors with IF1 positive expression were associated with a worse overall survival rate in glioma patients (P<0.05; Fig. 2). Notably, IF1 expression was an independent factor for predicting the overall survival rate of patients with glioma (P=0.018; Table II). These data indicate that IF1 may act as a potent biomarker for predicting the prognosis of glioma patients.

#### IF1 knockdown inhibits glioma cell migration and invasion

**Figure 3.**
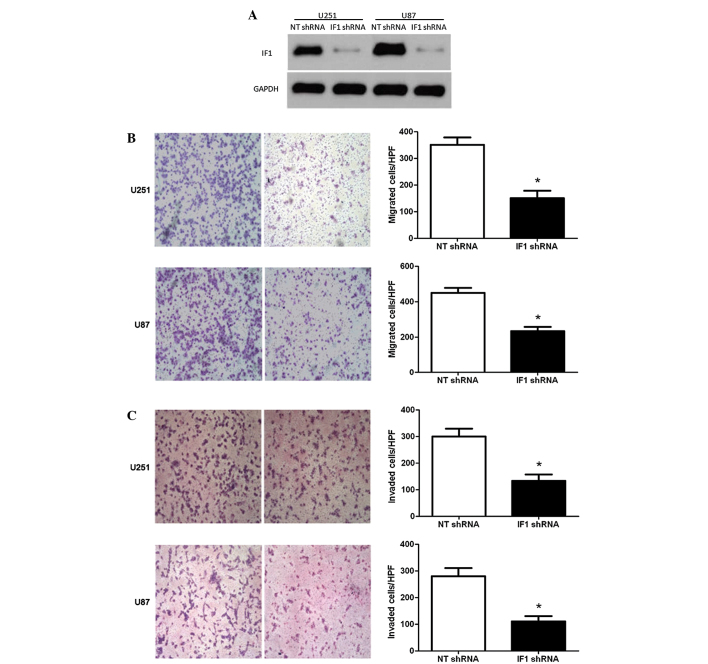
IF1 knockdown suppresses the migration and invasion of glioma cells. (A) U251 and U87 cells transfected with NT shRNA or IF1 shRNA were analyzed using western blotting to detect IF1 expression. Experiments were repeated in triplicate, with similar results. (B) Cell migration was measured by Transwell assays and was inhibited by IF1 knockdown in U251 and U87 cells, as compared with control cells (n=6). *P<0.05 vs. control. (C) Downregulation of IF1 in U215 and U87 cells conferred a decreased number of invaded cells compared with control cells (n=6). *P<0.05 vs. control. IF1, adenosine triphosphatase inhibitory factor 1; NT, non-targeting; shRNA, short hairpin RNA; HPF, high power fields.

To confirm the role of IF1 in glioma, U251 and U87 cells were transduced with NT or IF1 shRNA and then subjected to Transwell assays for cell migration and invasion. As measured by western blotting, the level of IF1 protein was significantly downregulated by specific shRNA in U251 and U87 cells (P<0.05; Fig. 3A). Transwell assays were performed to determine the effect of altering IF1 levels on tumor cell migration. IF1 knockdown resulted in a significant reduction of U251 and U87 cell migration (P<0.05; Fig. 3B). Furthermore, as determined by Transwell assays, the number of invasive U251 and U87 cells was prominently decreased subsequent to IF1 knockdown (P<0.05; Fig. 3C). Thus, IF1 may exert a pro-metastatic effect by promoting cell migration and invasion in glioma.

#### IF1 may promote glioma metastasis through the NF-κB/Snai1 pathway

**Figure 4.**
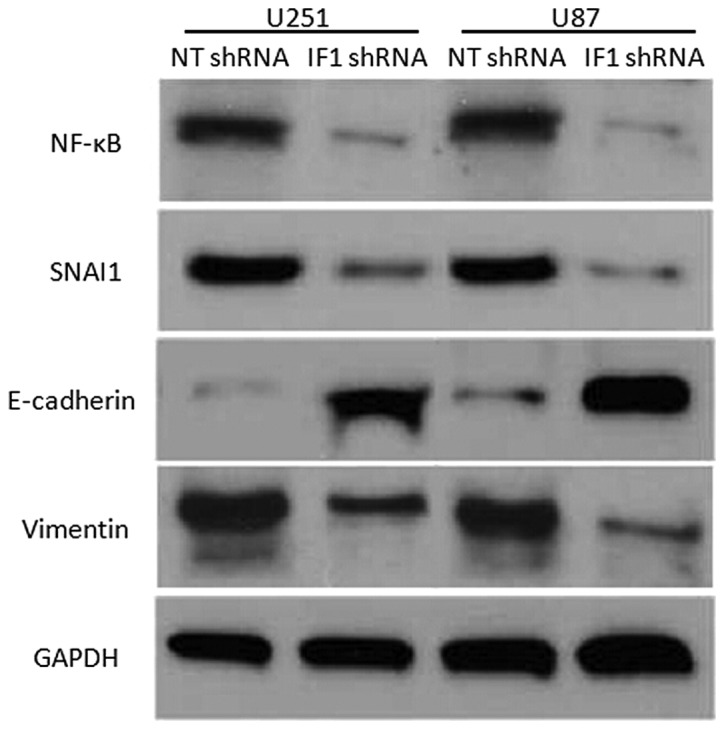
IF1 knockdown inhibited the NF-κB/Snai1 pathway in glioma cells. The U251 and U87 cells that were transduced with NT shRNA or IF1 shRNA were analyzed using western blotting to detect NF-κB, Snai1, E-cadherin and vimentin expression. Representative western blotting revealed that IF1 knockdown downregulated NF-κB, Snai1 and vimentin expression, but upregulated E-cadherin expression. Data are representative of multiple repeats with similar results. IF1, adenosine triphosphatase inhibitory factor 1; NT, non-targeting; shRNA, short hairpin RNA; NF-κB; nuclear factor-κB.

A previous study has reported that IF1 promotes HCC metastasis and angiogenesis through the NF-κB/Snai1 and vascular endothelial growth factor pathway (11). To investigate the potential signaling pathways involved in IF1 induced glioma cell migration and invasion, U251 and U87 cells that were transduced with NT shRNA or IF1 shRNA were subjected to western blot analysis for the expression of NF-κB, Snai1, E-cadherin and vimentin. Notably, IF1 knockdown inhibited NF-κB, Snai1 and vimentin expression, but upregulated E-cadherin expression (Fig. 4). Previous studies reported that NF-κB transcriptionally regulated Snai1 expression and subsequently promoted epithelial-mesenchymal transition (EMT). Thus, the present results indicate that IF1 may promote glioma metastasis via the NF-κB/Snai1 signaling pathway.

## Discussion

IF1 specifically inhibits the ATP-hydrolyzing activity of F1F0-ATP synthase, without impacting the synthesis of ATP during oxidative phosphorylation (16,17). Elevated expression of IF1 has been observed in numerous human cancers (9–11). IF1 has previously been considered to interact with the canonical NF-κB signaling pathway to promote tumor progression (9–11). In the present study, the expression of IF1 was detected in 86 glioma tissues and 20 NB tissues using immunohistochemical staining. It was found that IF1 protein expression in the glioma tissues was markedly increased compared with the expression in the NB tissues. In addition, the results of RT-qPCR indicated that the difference in IF1 mRNA expression between the glioma and NB tissues was consistent with IF1 protein expression. Clinical analysis revealed that the positive expression of IF1 was evidently associated with an advanced clinical stage of glioma. Notably, the present data revealed that the presence of IF1 expression conferred a significantly reduced overall survival rate for patients with glioma. Multivariate Cox regression analysis indicated that IF1 was an independent factor for predicting the overall survival of patients with glioma. Overall, the present results indicate that IF1 may be a potential oncogene and act as a prognostic biomarker for predicting the survival of glioma patients.

IF1 has previously been considered to be a crucial factor in tumorigenesis (9–11). However, the roles of IF1 and the IF1-mediated signaling pathways in glioma have yet to be elucidated. In the present study, a novel role of IF1 in glioma was revealed. IF1 was knocked down in U251 and U87 cells through the transfection of exogenous shRNA. Transwell cell migration assays found that IF1 knockdown led to a significant reduction in the cell migration of U251 and U87 cells. In addition, the Transwell cell invasion assays revealed that IF1 knockdown decreased the number of invasive U251 and U87 cells. The present data indicated that IF1 may promote tumor progression by promoting migration and invasion in glioma cells. An increase in NF-κB signaling is a key tumor survival mechanism, and promotes processes involved in tumor metastasis, including EMT, resistance to apoptosis and angiogenesis (18). EMT is a dynamic and reversible cellular process that is characterized by the loss of cell polarity and intracellular junctions and the acquirement of mesenchymal features, resulting in increased cell migration and invasion (19). Cancer cells that undergo EMT lead to tumor metastasis and poor survival in patients (20). Previous studies have reported that glioma cells with EMT exhibit enhanced invasion and metastatic potential (21,22). Furthermore, tumor tissues that were obtained from glioma patients were used for molecular subtyping, and the data indicated that tumors with mesenchymal gene characteristics conferred a reduced overall survival rate and resistance to treatment in patients, indicating that EMT plays a key role in the progression of glioma (19). In the present study, IF1 knockdown inhibited the expression of NF-κB and Snai1, a key regulator of EMT. Furthermore, IF1 knockdown led to the increased expression of E-cadherin and reduction of vimentin expression. The current data suggest that the NF-κB/Snai1 axis may be responsible for IF1-mediated metastasis in glioma.

In conclusion, the present study found that the expression of IF1 is elevated in glioma tissues and the presence of IF1 expression is associated with an advanced clinical stage. Furthermore, IF1 is an independent prognostic marker for predicting the overall survival of glioma patients. IF1 knockdown decreases the number of migratory and invasive glioma cells in glioma tissue. The NF-κB/Snai1 axis may be involved in the IF1-mediated metastasis of glioma. Overall, IF1 may be a potential valuable biomarker and therapeutic target in human glioma.

## Figures and Tables

**Table I.** Association between clinicopathological characteristics of glioma patients and expression of the IF1 protein (n=86).

		IF1 protein expression		
			
Characteristics	Total, n	Present, n	Absent, n	P-value
Age (years)				
<50	40	29	11	0.468
≥50	46	30	16	
Gender				
Male	55	38	17	0.897
Female	31	21	10	
Histological type				
Astrocytic tumors	62	42	20	0.153
Oligodendrogial tumors	9	7	2	
Oligoastrocytic tumors	15	10	5	
WHO grade				
I+II	26	12	14	0.003
III+IV	60	47	13	

WHO, World Health Organization; IF1, adenosine triphosphatase inhibitory factor 1.

**Table II.** Multivariate Cox regression analysis of the overall survival time of glioma patients.

Variables	HR	95% CI	P-value
Age	2.113	0.888–5.030	0.091
Gender	0.749	0.339–1.652	0.474
Histological type	1.846	0.694–4.908	0.219
WHO grade	3.576	1.251–10.211	0.017
IF1 expression	0.253	0.081–0.790	0.018

WHO, World Health Organization; IF1, adenosine triphosphatase inhibitory factor 1; HR, hazard ratio; CI, confidence interval.
